# Post-Levothyroxine Thyroid Dysfunction in Saudi Arabian Patients with Hypothyroidism: A Cross-Sectional Study

**DOI:** 10.3390/clinpract16060116

**Published:** 2026-06-17

**Authors:** Baraah Ghssan AlHassan, Maujid Masood Malik, Ahmed Mohamedain, Adnan Jehangir, Farhana Ayub, Omer Musa, Ahmed Ibrahim, Habib Ahmad Qureshi, Hayder A. Giha

**Affiliations:** 1 Department of Biomedical Sciences, College of Medicine, King Faisal University, Al-Ahsa P.O. Box 400, Saudi Arabia; 222403167@student.kfu.edu.sa (B.G.A.); drmaujid@hotmail.com (M.M.M.); amalik@kfu.edu.sa (A.J.); drfarhanaayubbiochem@gmail.com (F.A.); omermusa12@gmail.com (O.M.); 2 Department of Public Health, College of Applied Medical Sciences, King Faisal University, Al-Ahsa 31982, Saudi Arabia; aimohamed@kfu.edu.sa; 3 Mohammed Al-Mana College for Medical Sciences, Al Khobar 34222, Saudi Arabia; h.qureshi@machs.edu.sa; 4 Centre for Cardiovascular Science, University of Edinburgh, 47 Little France Crescent, Edinburgh EH16 4TJ, UK

**Keywords:** hypothyroidism, thyroid stimulating hormone, L-T4, vitamin D, obesity, dyslipidemia

## Abstract

Background: Post-thyroxine treatment of thyroid dysfunction remains a clinical concern, especially in Middle Eastern populations. Methods: This descriptive cross-sectional study was conducted in 2023 at King Fahad Hospital, Hufof, Kingdom of Saudi Arabia. Of the 237 patients treated with L-thyroxine (L-T4) for hypothyroidism, 163 patients, almost exclusively females (152 females, 11 males), met the inclusion criteria and were enrolled. Thyroid hormones, lipid profiles, and 25-hydroxyvitamin D (25OH-D) were measured using standard laboratory assays. Results: Only 57% of patients achieved euthyroid status following L-T4 treatment, while 12.3% developed post-thyroxine-treatment (PTT) hyperthyroidism, and 30.7% developed PTT hypothyroidism. Older age was significantly associated with dysthyroidism (*p* = 0.018), whereas obesity (*p* = 0.937) and vitamin D levels (*p* = 0.982) were not. Total cholesterol (TC) and LDLc positively correlated with TSH levels, while elevated triglycerides (TGs) were significantly associated with PTT hyperthyroidism. The two dysthyroid subgroups were comparable across all non-thyroid parameters, including age, BMI, 25(OH)D levels, and lipid fractions. However, free T4 was significantly higher in PTT hyperthyroidism (*p* < 0.001); free T3 showed a trend toward higher levels in PTT hyperthyroidism (*p* = 0.052); and TSH was significantly higher in PTT hypothyroidism (*p* < 0.001). Conclusions: The proportions of patients with PTT hypo- and hyperthyroidism are aligned with international observations. Furthermore, the age was significantly associated with dysthyroidism, and dyslipidemia is the most consistent biochemical correlate of suboptimal thyroid status; however, the associations of PTT dysthyroidism with hypovitaminosis D and BMI were not noticed in this setting.

## 1. Introduction

Globally, hypothyroidism is a common condition with a prevalence that is significantly higher in women than in men, often increasing in frequency with advancing age (Vanderpump et al., 1995) [1]. In women, the mean age at the time of diagnosis is approximately 60 years, primarily driven by the high incidence of chronic autoimmune thyroiditis (Chaker et al., 2017) [2]. In the Kingdom of Saudi Arabia (KSA), the burden of thyroid dysfunction is particularly pronounced; recent meta-analyses indicate a national prevalence of approximately 31.3%, with subclinical hypothyroidism (SCH) accounting for nearly 18.9% of these cases (Alhajri et al., 2025, Kargar et al., 2024) [3,4]. Despite this high prevalence, regional management remains a challenge, with studies in Saudi primary care settings identifying significant gaps in treatment optimization and dose titration (Alluhayyan et al., 2020) [5].

The physiological regulation of thyroid hormones (TH) relies on a complex homeostatic loop: the hypothalamus secretes thyrotropin-releasing hormone (TRH) to stimulate the pituitary’s release of a thyroid-stimulating hormone (TSH), which in turn governs the production of thyroxine (T4) and triiodothyronine (T3) by the thyroid gland. This system is maintained via a negative feedback mechanism where elevated levels of free T3 (fT3) or free T4 (fT4) suppress further TSH and TRH secretion (Chiamolera and Wondisford, 2009) [6]. When this equilibrium is disrupted, patients manifest classic clinical symptoms, such as fatigue, weight gain, cold intolerance, and thick skin (Wilson et al., 2021, Zamwar and Muneshwar, 2023) [7,8]. However, a significant subset of the population presents with “subclinical” dysfunction, defined by elevated TSH levels despite normal circulating fT4/fT3. This condition affects up to 15% of adults and is increasingly recognized as a modifiable risk factor for metabolic and cardiovascular disorders (Tseng et al., 2012, Suh and Kim, 2015) [9,10].

Beyond the primary symptoms, hypothyroidism is intrinsically linked to metabolic dysfunction. Thyroid hormones are essential regulators of energy expenditure, lipid clearance, and the conversion of cholesterol into bile acids (Mullur et al., 2014) [11]. Consequently, even mild elevations in TSH have been associated with adverse lipid profiles, including increased total cholesterol (TC) and low-density lipoprotein (LDL) levels (Liu et al., 2014) [12]. Standard levothyroxine (L-T4) monotherapy aims to normalize these markers by providing a pro-hormone for peripheral conversion into active T3. Yet, it is estimated that 5–10% of patients remain symptomatic or exhibit suboptimal metabolic control even with “normalized” TSH (Wiersinga et al., 2012) [13]. This persistent dysfunction may be attributed to genetic polymorphisms in deiodinase enzymes or environmental deficiencies that impair the intracellular activation of T4.

Emerging evidence suggests that vitamin D has been proposed as a modulator of peripheral T4-to-T3 conversion, though the evidence remains inconsistent. The active form of vitamin D may upregulate the expression of type 2 deiodinase (DIO2), the primary enzyme responsible for converting T4 into the more metabolically active T3 in peripheral tissues (Duntas & Brenta, 2012, Vassalle et al., 2021) [14,15]. Given the high prevalence of both vitamin D deficiency (Al-Alyani et al., 2018) [16] and thyroid dysfunction (Alqahtani, 2021) [17] in the country, understanding the interplay between these two factors is critical for improving clinical outcomes. This study aims to estimate the rate of post-thyroxine treatment (PTT) dysthyroidism and its association/correlation with obesity and hypovitaminosis in an Arab cohort, and to elucidate the impact of this hormonal imbalance on the systemic lipid profile.

## 2. Materials and Methods

Study design and setting: This descriptive cross-sectional study was conducted between June and August 2023 in the Kingdom of Saudi Arabia (KSA). The research was conducted at the Endocrinology and Diabetes Centre at King Fahad Hospital, Hufof (KFHH). In this hospital, as in the rest of the KSA hospitals, the diagnosis and management of hypothyroidism and patient follow-up are based on the national protocol, which is adapted from the USA and United Kingdom thyroid societies (Alsifri et al., 2022) [18]. The initial diagnosis of hypothyroidism is based mainly on TSH measurement, in addition to fT3 and fT4, and several other investigations to determine the cause of hypothyroidism to defined the exact cause of hypothyroidism (Alzahrani et al., 2020) [19]. During the follow-up, the patients are advised to use the thyroid treatment (L-T4) after the morning blood sampling, which is used for testing the thyroid and other biochemical parameters (Alsifri et al., 2022) [18].

Study Population and Selection: The study targeted adult patients diagnosed with hypothyroidism. Inclusion criteria comprised males and females aged 18 years and older residing in Al-Ahsa. Exclusion criteria included patients under 18, those with co-existing chronically severe diseases that required lifelong or prolonged therapy (e.g., complicated T2D or hypertension, chronic kidney diseases, chronic heart diseases, cancer, tuberculosis, psychiatric diseases, and neurodegenerative diseases), pregnant or lactating women, and individuals taking medications known to interfere with L-T4 therapy or affect lipid metabolism. Out of an initial screening of 1260 suspected patients, 237 were identified with hypothyroidism, and 208 met the primary inclusion criteria. After excluding those with incomplete routine laboratory evaluations, a final cohort of 163 patients (152 females and 11 males) was enrolled.

All participants were receiving levothyroxine (L-T4) therapy at the time of the study. However, the data are missing the detailed clinical information relevant to therapy, namely, the levothyroxine dose, dose per body weight, duration of therapy and adherence, duration since diagnosis, and timing of blood sampling relative to levothyroxine intake. In addition, the etiology of hypothyroidism, thyroid autoantibody status, prior thyroid surgery or radioiodine treatment, and use of medications or supplements that may interfere with absorption, such as calcium, iron, proton pump inhibitors, bile acid sequestrants, or anticonvulsants, are also missing. This is a major limitation in this study that needs to be considered in the interpretation of our findings.

Data Collection: Demographic information and laboratory data were extracted and recorded in Microsoft Excel. Informed consent was obtained and signed by all participants before their inclusion in the study.

Ethical Considerations: The study protocol received ethical approval from the Research Committee at the College of Medicine, King Faisal University, the Institutional Review Board (IRB) (H-05-HS-065), and the Ethical Committee for Scientific Research at King Fahad Hospital.

### 2.1. Biochemical Analysis

All biochemical analyses were performed at the clinical laboratory of King Fahad Hospital, Hufof (KFHH), using automated platforms to ensure high analytical precision.

Lipid profile estimation: Serum samples were analyzed using a Roche Cobas automated clinical chemistry analyzer (Roche Diagnostics, Mannheim, Germany). Total cholesterol (TC), triglycerides (TGs), and high-density lipoprotein cholesterol (HDLc) were determined via direct enzymatic colorimetric methods. Low-density lipoprotein cholesterol (LDLc) was calculated using the Friedewald formula: LDLc = TC − HDLc − (TG/2.2). All lipid parameters were recorded in mmol/L.

Thyroid function tests: Thyroid-stimulating hormone (TSH), free thyroxine (fT4), and free triiodothyronine (fT3) were quantified using the electrochemiluminescence immunoassay (ECLIA) technique on the Roche Cobas e-series/Elecsys immunoassay analyzer (Roche Diagnostics GmbH, Mannheim, Germany). TSH levels were reported in micro-international units per litre (µIU/mL). fT4 and fT3 levels were reported in picomoles per litre (pmol/L).

Vitamin D estimation: Total serum 25-hydroxyvitamin [25(OH)D] was measured using a competitive ECLIA binding assay on the Roche Cobas e-series platform. This assay provides a quantitative determination of total vitamin D status (including both D2 and D3 fractions). Results were expressed in ng/mL.

### 2.2. Statistical Analysis

Data were analyzed using SigmaStat (version 3.5, Systat Software Inc., San Jose, CA, USA). Continuous variables were expressed as mean ± standard deviation (SD) or median (interquartile range) based on their distribution. For group comparisons, the independent *t*-test or one-way Analysis of Variance (ANOVA) was used for normally distributed data. For non-parametrically distributed variables, the Mann–Whitney U test or Kruskal–Wallis test was applied. Dunn’s method was used for all Pairwise Multiple Comparison Procedures. Specifically, these tests were used to compare: euthyroidism vs. dysthyroidism; hyperthyroidism vs. hypothyroidism; hyperthyroidism vs. euthyroidism vs. hypothyroidism; and TSH concentrations across quartiles (Q1, Q2, Q3, and Q4). Pearson’s correlation coefficient was employed to evaluate the strength and direction of associations between TSH and all other clinical and biochemical variables. A *p*-value ≤ 0.05 was defined as the threshold for statistical significance.

## 3. Results

### 3.1. Characteristics of the Study Subjects

One hundred and sixty-three patients with hypothyroidism on levothyroxine (L-T4) replacement therapy were included. Their thyroid hormones, vitamin D, and anthropometric profiles, including sex and age, are presented in Table 1.

Regarding thyroid function, most patients had fT3 within the normal reference range (95.1%), with 3.1% showing subnormal and 1.8% above-normal values, respectively. fT4 was within reference range in 73.6% of patients, subnormal in 23.9%, and above normal in 2.5%. TSH was within the normal range in 57.0%, elevated in 30.7%, and suppressed in 12.3%.

Vitamin D insufficiency and deficiency were highly prevalent: 32.7% of patients had hypovitaminosis D (insufficiency 29%; deficiency 3.7%), and no patient had an abnormally elevated 25(OH)D level. Regarding BMI, 55.8% of patients were classified as obese, 40.5% were non-obese (normal and overweight), and 3.4% were underweight.

### 3.2. Classification of Patients by Post-Treatment TSH Status

Although all patients were attending routine follow-up with no or minimal clinical symptoms, biochemical TSH levels varied considerably. Patients were therefore stratified into three post-thyroxine treatment (PTT) groups: i. PTT hyperthyroidism (TSH < 0.5 µIU/mL; n = 20, 12.3%), including one patient with high fT3, two patients with high fT4, and two patients with low fT4 [35 years old female, and 64 years old female]. ii. Euthyroid (TSH 0.5–5.0 µIU/mL; n = 93, 57.0%), including one patient with high fT3, one patient with low fT3, and 15 patients with low fT4. iii. PTT hypothyroidism (TSH > 5.0 µIU/mL; n = 50, 30.7%), including one patient with high fT3 [50 years old female], two patients with low fT3, two patients with high fT4 [22 years old female, and 29 years old male], and 22 patients with low fT4) (Table 2). Notably, both clinical data and fT3 (active form) largely support the TSH-based classification (Table 2). For exploration analysis, the patients were stratified into four groups by TSH quartile: Q1 (n = 40), Q2 (n = 41), Q3 (n = 41), and Q4 (n = 41).

### 3.3. Comparison of Clinical and Biochemical Parameters Across PTT Groups

Thyroid hormones: fT4 was significantly higher in PTT hyperthyroidism patients than in euthyroid and PTT hypothyroidism patients (17.26 [14.31–19.48] vs. 14.63 [12.70–16.52] vs. 12.67 [11.10–15.21] pmol/L; *p* < 0.001), consistent with the inverse relationship between TSH and fT4 in patients on L-T4. fT3 showed a non-significant trend toward higher values in PTT hyperthyroidism (4.92 [3.82–5.34] vs. 4.14 [3.80–4.67] vs. 4.17 [3.80–4.73] pmol/L; *p* = 0.089).

Lipid profile: TG levels were significantly higher in PTT hyperthyroidism compared with euthyroid patients (1.49 [1.17–2.00] vs. 1.01 [0.76–1.64] mmol/L; *p* = 0.022, Kruskal–Wallis test). This elevation was driven specifically by the contrast between PTT hyperthyroidism and euthyroid patients; the pairwise comparison between PTT hyperthyroidism and PTT hypothyroidism was not significant (1.26 [1.01–1.59] mmol/L; *p* = 0.078, Mann–Whitney test). Total cholesterol, LDLc, and HDLc did not differ significantly across the three groups (*p* = 0.333, 0.183, and 0.679, respectively).

Age, BMI, and vitamin D: PTT hyperthyroidism patients were significantly older than euthyroid and PTT hypothyroidism patients (45.00 [39.25–52.25] vs. 38.00 [33.50–45.50] vs. 41.50 [33.75–48.50] years; *p* = 0.018). BMI and 25(OH)D were comparable across all three groups (*p* = 0.937 and *p* = 0.982, respectively).

### 3.4. Euthyroid Versus Dysthyroid Patients

**Figure 1:**
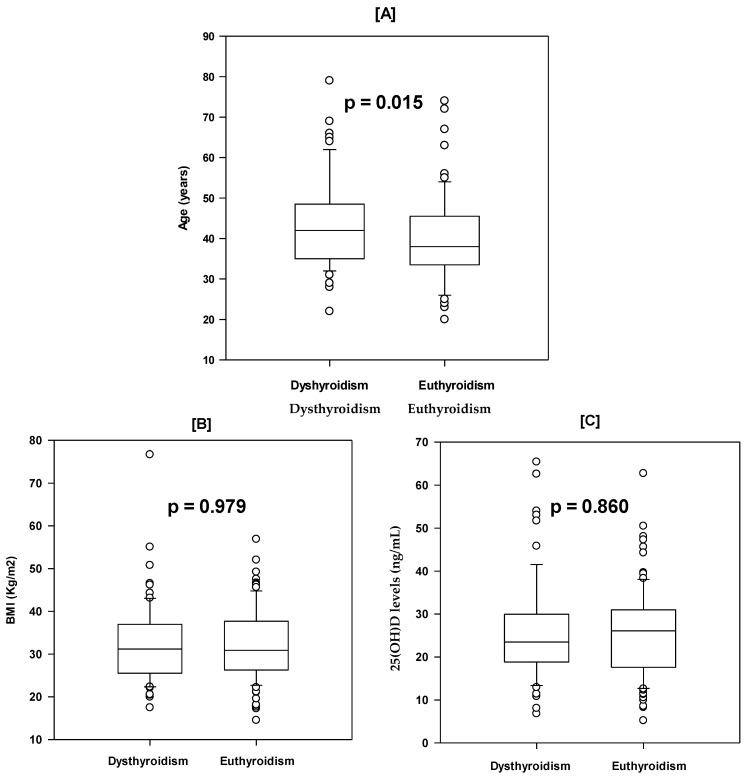
Comparison of age, BMI, and vitamin D status between dysthyroidism and euthyroidism groups: Box plots comparing the distribution of clinical parameters between individuals with dysthyroidism and euthyroidism. The horizontal line within each box represents the median, box boundaries indicate the interquartile range (IQR), whiskers extend to 1.5× the IQR, and open circles denote outliers. (**A**) Age (years) was significantly higher in the dysthyroidism group compared to the euthyroidism group (*p* = 0.015). (**B**) BMI showed no significant difference between the two groups (*p* = 0.979). (**C**) Serum 25(OH)D levels (ng/mL) did not differ significantly between the dysthyroidism and euthyroidism groups (*p* = 0.860).

When PTT hyper- and hypothyroidism patients were combined into a single dysthyroid group and compared with euthyroid patients, the euthyroid group was significantly younger (38.00 [33.50–45.50] vs. 42.00 [35.00–48.50] years; *p* = 0.015). BMI and 25(OH)D did not differ between groups (30.87 [26.28–37.71] vs. 31.19 [25.52–36.97] kg/m^2^, *p* = 0.979; and 26.09 [17.59–30.98] vs. 23.48 [18.84–29.96] µg/mL, *p* = 0.860, respectively) (Figure 1).

### 3.5. Comparison Between PTT Hyperthyroidism and PTT Hypothyroidism

Direct comparison of the two dysthyroid groups revealed that TSH was significantly higher in PTT hypothyroidism (8.24 [6.51–12.44] vs. 0.076 [0.013–0.290] µIU/mL; *p* < 0.001), while fT4 was significantly higher in PTT hyperthyroidism (17.26 [14.31–19.48] vs. 12.67 [11.10–15.21] pmol/L; *p* < 0.001). fT3 was borderline (*p* = 0.052), and age, BMI, 25(OH)D, and all lipid parameters were comparable between the two groups (*p* = 0.145, 0.706, 0.926, 0.178, 0.101, 0.903, and 0.078, respectively) (Table 2).

### 3.6. Correlation Analyses

**Figure 2:**
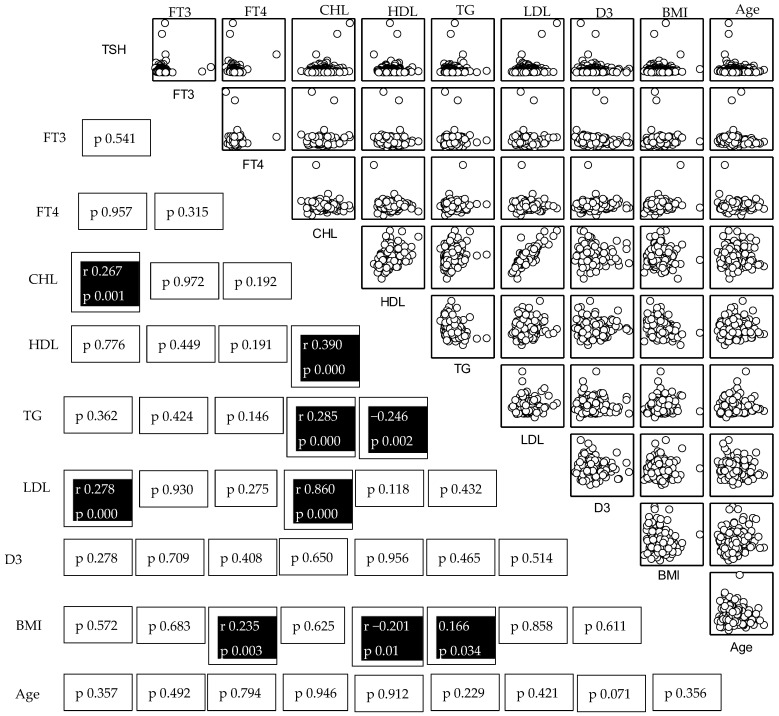
Correlation matrix of thyroid function test parameters and clinical/metabolic variables: The figure displays pairwise correlations among thyroid function parameters, TSH, fT3, and fT4, and metabolic/clinical variables, including TC, HDLc, TG, LDLc, 25(OH)D, BMI, and age. The upper triangle shows scatter plots for each variable pair. The lower triangle displays Pearson correlation coefficients (r) and corresponding *p*-values for each pair. Statistically significant correlations (*p* < 0.05) are highlighted with a black background. Notable significant associations include: TSH with TC (r = 0.267, *p* = 0.001) and LDLc (r = 0.278, *p* < 0.001); TC with HDLc (r = 0.390), TG (r = 0.285), and LDLc (r = 0.860); HDLc with TG (r = −0.246, *p* = 0.002); fT4 with BMI (r = 0.235, *p* = 0.003); and BMI with HDLc (r = −0.201, *p* = 0.010) and TG (r = 0.166, *p* = 0.034). Note: CHL = TC (total cholesterol); D3 = 25(OH)D (vitamin D). Note: the open circles stand for each study subject separately, and the black background highlight the significant correlations.

TSH and lipid profile: TSH was significantly and positively correlated with TC (r = 0.267, *p* = 0.001) and LDLc (r = 0.278, *p* < 0.001). No significant correlation was found between TSH and HDLc or TG. HDLc and TG were inversely correlated with each other (r = −0.248, *p* = 0.001) (Figure 2). TSH, thyroid hormones, vitamin D, and BMI: TSH showed no significant correlation with fT3 (r = 0.048, *p* = 0.541), fT4 (r = 0.004, *p* = 0.957), 25(OH)D (r = −0.086, *p* = 0.278), or BMI (r = 0.045, *p* = 0.572). fT4, fT3, and BMI: fT4 was positively correlated with BMI (r = 0.235, *p* = 0.003). fT3 was not significantly correlated with any parameter examined (Figure 2). BMI and lipid profile: BMI was positively correlated with TG (r = 0.166, *p* = 0.034) and negatively correlated with HDLc (r = −0.201, *p* = 0.010). Neither age nor 25(OH)D was significantly correlated with any of the parameters tested (Figure 2). TC was strongly and positively correlated with LDLc (r = 0.860, *p* < 0.001), HDLc (r = 0.390, *p* < 0.001), and TG (r = 0.285, *p* < 0.001), in addition to its correlation with TSH noted above.

### 3.7. TSH Quartile Analysis

Age, BMI, and vitamin D: Age, BMI, and 25(OH)D did not differ significantly across TSH quartiles (*p* = 0.371, 0.413, and 0.540, respectively), with BMI ranging from 30.48 (25.29–37.41) to 32.88 (26.52–40.23) kg/m^2^, age from 38.00 (30.00–46.00) to 42.00 (35.25–47.00) years, and 25(OH)D from 23.44 (18.42–29.26) to 28.02 (19.81–30.70) ng/mL (Table 3).

Thyroid hormones: TSH values across quartiles were: Q1 (0.48 [0.07–0.76]), Q2 (1.92 [1.45–2.20]), Q3 (3.80 [3.15–4.98]), and Q4 (9.78 [7.69–14.89]) µIU/mL (*p* < 0.001). fT4 showed a significant inverse trend across quartiles: Q1 (16.17 [14.25–18.88]), Q2 (14.00 [12.38–16.38]), Q3 (14.03 [11.83–15.70]), and Q4 (12.76 [11.06–16.10]) pmol/L (*p* < 0.001). fT3 did not differ across quartiles (*p* = 0.689). Lipid profile: TC differed significantly across TSH quartiles (*p* = 0.036), with the lowest values in Q1 and Q3 and the highest in Q2 and Q4, a non-monotonic pattern. Similarly, LDLc differed significantly across quartiles (*p* = 0.011), with the lowest values in Q1 and Q3 and the highest in Q2 and Q4 (Table 3; Kruskal–Wallis test).

## 4. Discussion

This study demonstrates that a substantial proportion of hypothyroid patients receiving L-T4 replacement therapy fail to achieve optimal biochemical control, with only 57.0% reaching euthyroidism, representing a nonoptimal management rate of 43.0%. This is of particular clinical relevance, given that this cohort was drawn from a region of endemic hypothyroidism in Saudi Arabia (Alqahtani et al., 2021, Alhajri et al., 2025) [3,17] and is broadly consistent with treatment outcome data reported in other populations (Gunasekaran et al., 2024) [20]. Of those who did not achieve euthyroidism, 30.7% had elevated TSH, classified here as post-thyroxine treatment (PTT) hypothyroidism, while 12.3% had suppressed TSH, classified as PTT hyperthyroidism. Although the clinical features at presentation and the specific symptoms and signs following L-T4 treatment were not systematically recorded, no serious adverse symptoms were reported at the time of assessment. These findings underscore the need for routine biochemical surveillance in all L-T4-treated patients, independent of symptomatic status.

Four factors have previously been identified as risk factors for thyroid disease and suboptimal treatment response: older age, male sex, obesity, and vitamin D deficiency (Gunasekaran et al., 2024; Duntas et al., 2019, Łukawska-Tatarczuk et al., 2024, Feldt-Rasmussen et al., 2024) [20,21,22,23]. In the present study, age was the only factor that significantly differentiated euthyroid from dysthyroid patients: those who achieved euthyroidism were significantly younger than those who did not (38.00 vs. 42.00 years; *p* = 0.015). Age-related changes in L-T4 absorption, intestinal motility, body composition, or tissue sensitivity to thyroid hormones (Duntas et al., 2019) [21] may contribute to this observation, though the modest absolute age difference between groups warrants cautious interpretation.

BMI did not differ significantly between euthyroid and dysthyroid patients, nor between PTT hyperthyroid and PTT hypothyroid subgroups, which is at variance with prior reports linking obesity to suboptimal L-T4 replacement (Mele et al., 2019) [24]. The high overall prevalence of obesity in this cohort (55.8%) may have attenuated the discriminatory power of BMI as a between-group variable. However, sex data were uninformative in this study, as males comprised only 11 of 163 patients (6.7%), precluding meaningful sex-stratified analysis; the established association between male sex and thyroxine under-replacement (Łukawska-Tatarczuk et al., 2024; Gunasekaran et al., 2024) [20,23] could therefore neither be confirmed nor refuted in this dataset.

The association between vitamin D deficiency and hypothyroidism is well established (Appunni et al., 2021) [25], with proposed mechanisms, including immunomodulatory suppression of anti-thyroid peroxidase autoantibodies, a primary driver of autoimmune hypothyroidism, through vitamin D receptor (VDR)-mediated signaling (Sun et al., 2025) [26]. Population-level vitamin D screening has accordingly been proposed as a strategy to mitigate hypothyroidism risk (Appunni et al., 2021) [25]. Despite the high prevalence of hypovitaminosis D in this cohort (32.7%), 25(OH)D levels were comparable between euthyroid and dysthyroid patients, between PTT hyperthyroid and PTT hypothyroid subgroups, and across all TSH quartiles, with no significant correlation identified between 25(OH)D and TSH (r = −0.086, *p* = 0.278). This null finding is consistent with the recent work of Babić Leko et al. (2023) [27], who reported either a negative correlation or the absence of any association between TSH and 25(OH)D across multiple study populations. A mechanistically plausible explanation is inter-individual variation in VDR gene polymorphisms, which may modulate the functional cellular response to circulating vitamin D independently of serum 25(OH)D concentration. VDR polymorphism is prevalent in this population and has previously been associated with obesity (Al-Daghri et al., 2017) [28], suggesting that genetic variation in vitamin D signaling may confound or modify any relationship between vitamin D status and thyroid function in this setting.

Dyslipidemia is a well-recognized biochemical consequence of thyroid dysfunction and a clinically relevant biomarker of suboptimal thyroid hormone status (Duntas and Brenta, 2012) [14]. In the present study, TSH was significantly and positively correlated with TC (r = 0.267, *p* = 0.001) and LDLc (r = 0.278, *p* < 0.001), consistent with the atherogenic lipid phenotype associated with hypothyroid states. Although raised TGs are physiologically more associated with hypothyroidism, some studies reported a slight but significant increase in hyperthyroidism (Nikkilä et al., 1972; Sigal et al. 2020, Asemota, et al., 2021; Lee et al., 2023) [29,30,31,32]. In the present study, the TG levels were significantly elevated in PTT hyperthyroidism compared with euthyroid patients (*p* = 0.022), a finding that may reflect increased hepatic lipogenesis or reduced lipoprotein lipase activity under conditions of relative thyroid hormone excess (Liu et al., 2014) [12]. In contrast, TC, LDLc, and HDLc did not differ significantly across the three PTT groups, suggesting that TG may represent a more sensitive early biochemical marker of lipid perturbation in dysthyroidism than the conventional cholesterol fractions in this population. The inverse correlations between HDLc and TG (r = −0.248, *p* = 0.001) and between HDLc and BMI (r = −0.201, *p* = 0.010) are internally consistent with established metabolic relationships (Hussain et al., 2019) [33]. The positive correlation between fT4 and BMI (r = 0.235, *p* = 0.003) is also noteworthy and may reflect compensatory upregulation of thyroid hormone levels in the context of obesity-related metabolic demand, though this requires further investigation.

A recognized methodological challenge in studies of subclinical thyroid dysfunction is the reliance on population-derived TSH reference ranges, which are subject to considerable inter-laboratory and inter-population variability and may not adequately capture clinically meaningful biochemical gradients within a treated cohort (Garber et al., 2012, Kuś et al., 2024) [34,35]. To address this, TSH quartile stratification was employed as a complementary, threshold-independent approach to evaluate the associations between TSH and metabolic parameters. This analysis confirmed and extended the group-based findings: TC and LDLc differed significantly across TSH quartiles (*p* = 0.036 and *p* = 0.011, respectively), reinforcing the graded relationship between TSH and lipid metabolism even within a subclinical range (Li et al., 2021) [36]. Importantly, age, BMI, and 25(OH)D were uniformly distributed across quartiles, indicating that the lipid associations with TSH were independent of these confounders. The non-monotonic pattern of TC and LDLc across quartiles, with the lowest values in Q1 and Q3 and highest in Q2 and Q4, does not conform to a simple linear dose–response relationship, and it could be due to sampling variability. However, it may reflect the complex, non-linear interplay between TSH, free thyroid hormone levels, and lipid metabolism in a treated population, as recognized before (Li et al., 2024) [37]. This observation stands as a true statistical challenge in dealing with biological systems that are not following the dose–response relationship, and therefore, warrants further investigation in larger, prospectively designed studies.

A notable and unexpected finding was that fT3 levels were not significantly correlated with fT4, TSH, or any lipid parameter, did not differ significantly across PTT groups, and failed to discriminate between PTT hyper- and hypothyroidism. This apparent uncoupling from expected thyroid axis dynamics warrants further investigation, given that T3 is the primary biologically active thyroid hormone, generated predominantly through peripheral deiodination of T4 and subject to negative feedback regulation of TSH secretion (Van Uytfanghe et al., 2023) [38]. One mechanistic explanation is reduced or polymorphic type 2 deiodinase (DIO2) activity, which would impair T4-to-T3 conversion independently of circulating T4 levels. Emerging evidence suggests that DIO2 polymorphisms influence tissue T3 availability and may affect clinical and biochemical outcomes in L-T4-treated patients (Castagna et al., 2017, Jo et al., 2019, Mazza, 2025) [39,40,41]. Prospective studies incorporating deiodinase genotyping would be required to evaluate this hypothesis directly in this population. An alternative explanation for the observed dissociation of fT3 is analytical imprecision of the ECLIA fT3 assay at the lower-normal range in treated patients (Welsh and Soldin, 2016) [42], which cannot be excluded without assay-specific validation data.

Two patients from the PTT hyperthyroidism and three patients from the PTT hypothyroidis groups showed fT4 and fT3 levels inconsistent with their TSH profiles. From a research perspective, these may be considered “noisy observations” resulting from lab or data entry errors that do not alter the final analysis. Though the blood level of TSH is generally more stable and the L-T4 has a long half-life (approximately 1 week), the former is less likely to be affected by a missed single dose of fT4 or a pill taken less than 2 h before blood sampling (Colucci et al., 2013; Rajput & Pathak, 2017) [43,44]. However, improper sampling time remains as a possible explanation for the above controversy (Ain et al.,1993) [45]. Ultimately, these instances highlight why maintaining detailed clinical and medication histories is vital during patient follow-up. Importantly, although age can result in these contradictions between the levels of TSH and fT4/fT3 (Deary et al., 2012) [46], the ages of the five patients (22, 29, 35, 50, and 64 years, including four females and one male) were well distributed across the age range and sex of the study subjects.

The limitations of this study are as follows: (1) the cross-sectional design precludes causal inference; (2) a 2-month recruitment window may introduce seasonal vitamin D bias; (3) fT3 assay limitations as mentioned above; and (4). the lack of data about factors that affect L-T4 efficacy: levothyroxine dose, adherence, treatment duration, absorption-interfering medications, hypothyroidism etiology, and thyroid autoimmunity.

## 5. Conclusions

This study demonstrates that a substantial proportion of hypothyroid patients (43.0%) on L-T4 replacement therapy fail to achieve euthyroidism, with older age and dyslipidaemia serving as indicators of poor control in this setting. The sex, BMI, and vitamin D levels were uninformative in this study, which is likely reflecting the few numbers of males and the high background prevalence of obesity and hypovitaminosis D, respectively. To improve outcomes, future research should focus on investigating standardized, age-stratified dosing protocols, with consideration of obesity and hypovitaminosis, which are highly prevalent in the region.

## Figures and Tables

**Table 1:** Distribution of thyroid function tests, vitamin D status, and body mass index relative to reference ranges in L-T4-treated hypothyroid patients (n = 163).

Parameters (Unit)	Reference Range	Above Normal	Normal	Sub-Normal
TSH (µIU/mL)	0.5–5.0	30.7% (n = 50)	57% (n = 93)	12.3% (n = 20)
fT4 (pmol/L)	12–22	2.5% (n = 4)	73.6% (n = 120)	23.9% (n = 39)
fT3 (pmol/L)	3.1–6.8	1.8% (n = 3)	95.1% (n = 155)	3.1% (n = 5)
25(OH)D (ng/mL)	30–150	0% (n = 0)	67.3% (n = 109)	32.7% (n = 53) *
BMI (kg/m^2^)	18–<30	55.8% (n = 91)	40.5% (n = 66 [42 + 24])	3.4% (n = 6)

Footnote: * Of the 53 patients with hypovitaminosis D, 6 (3.7%) were vitamin D deficient (<20 ng/mL) and 47 (28.8%) were insufficient (20–29 ng/mL). Above normal and sub-normal TSH stands for elevated TSH (marker for hypothyroid function) and suppressed TSH (marker for hyperthyroid function). Normal BMI stands for normal and overweight subclasses of BMI together (non-obese).

**Table 2:** Demographic, biochemical, and lipid parameters stratified by post-thyroxine treatment thyroid status in hypothyroid patients on L-T4 therapy (n = 163).

Parameters	PTT-Hyperthyroidism	Euthyroid	PTT Hypothyroidism	*p* (All)	*p* (Hr vs. Ho)
Number (163)	12.3% (20)	57.0% (93)	30.7% (50)		
Sex (F/M)	19/1	87/6	46/4		
Age (years)	45.00 [39.25–52.25]	38.00 [33.50–45.50]	41.50 [33.75–48.50]	0.018	0.145
BMI (kg/m^2^)	32.23 [27.02–36.66]	30.87 [26.28–37.71]	30.93 [25.22–37.11]	0.937	0.706
25(OH)D (ng/mL)	23.95 [18.58–31.45]	26.09 [17.59–30.98]	23.48 [18.97–29.96]	0.982	0.926
fT4 (pmol/L)	17.26 [14.31–19.48]	14.63 [12.70–16.52]	12.67 [11.10–15.21]	<0.001	<0.001
fT3 (pmol/L)	4.92 [3.82–5.34]	4.14 [3.80–4.67]	4.17 [3.80–4.73]	0.089	0.052
TSH (µIU/mL)	0.076 [0.013–0.290]	2.10 [1.20–3.08]	8.24 [6.51–12.44]	<0.001	<0.001
TC (mmol/L)	4.50 [3.93–5.47]	4.90 [4.30–5.40]	5.08 [4.35–5.68]	0.333	0.178
LDLc (mmol/L)	2.58 [1.96–3.06]	2.86 [2.41–3.48]	3.00 [2.32–3.65]	0.183	0.101
HDLc (mmol/L)	1.29 [1.09–1.66]	1.40 [1.19–1.63]	1.36 [1.18–1.57]	0.679	0.903
TG (mmol/L)	1.49 [1.17–2.00]	1.01 [0.76–1.64]	1.26 [1.01–1.59]	0.022	0.078

Note: Hr = PTTS hyperthyroidism; Ho = PTTS hypothyroidism. Values expressed as median [IQR].

**Table 3:** Demographic, biochemical, and lipid parameters stratified by TSH quartile in L-T4-treated hypothyroid patients (n = 163).

	Q1 (Lowest TSH)	Q2	Q3	Q4 (Highest TSH)	*p*-Value
Number (163)	40	41	41	41	
Sex (F/M)	39/1	39/2	37/4	37/4	
Age (years)	42.00 [35.25–47.00]	38.00 [30.00–46.00]	39.00 [32.00–46.50]	41.00 [34.00–47.50]	0.371
BMI (kg/m^2^)	30.76 [26.29–34.76]	30.48 [25.29–37.41]	32.88 [26.52–40.23]	30.84 [25.13–36.50]	0.413
25(OH)D (ng/mL)	24.54 [18.80–31.98]	23.46 [14.90–31.59]	28.02 [19.81–30.70]	23.44 [18.42–29.26]	0.540
TSH (µIU/mL)	0.48 [0.07–0.76]	1.92 [1.45–2.20]	3.80 [3.15–4.98]	9.78 [7.69–14.89]	<0.001
fT4 (pmol/L)	16.17 [14.25–18.88]	14.00 [12.38–16.38]	14.03 [11.83–15.70]	12.76 [11.06–16.10]	<0.001
fT3 (pmol/L)	4.45 [3.78–5.14]	4.13 [3.80–4.61]	4.13 [3.87–4.83]	4.19 [3.79–4.75]	0.689
TC (mmol/L)	4.69 [4.40–5.48]	5.10 [4.53–5.77]	4.60 [3.91–5.27]	5.10 [4.38–5.74]	0.036
LDLc (mmol/L)	2.73 [2.22–3.33]	3.12 [2.71–3.69]	2.56 [2.13–3.27]	3.02 [2.37–3.75]	0.011
HDLc (mmol/L)	1.49 [1.21–1.75]	1.37 [1.14–1.67]	1.34 [1.10–1.46]	1.35 [1.19–1.58]	0.118
TG (mmol/L)	1.32 [0.82–1.69]	1.07 [0.82–1.94]	1.02 [0.83–1.56]	1.25 [1.00–1.61]	0.722

Values expressed as median [IQR]. *p*-values from Kruskal–Wallis test unless otherwise stated.

## Data Availability

The datasets presented in this article are not readily available due to technical/time limitations. Requests to access the datasets should be directed to the corresponding authors.
